# Marker-assisted screening of resistance to fire blight, powdery mildew, and apple scab in local apple varieties from Uzbekistan

**DOI:** 10.1371/journal.pone.0357988

**Published:** 2026-09-15

**Authors:** Abdushukur I. Rakhmatullaev, Muhabbat K. Turdieva, Marguba M. Rejapova, Abrorjon A. Abdurakhimov, Alisher A. Abdullaev

**Affiliations:** 1 Biotechnology Department, Center for Advanced Technologies, Almazar, Tashkent, Uzbekistan; 2 office for Central Asia, Alliance of Bioversity International and CIAT, Tashkent, Uzbekistan; Nuclear Science and Technology Research Institute, IRAN, ISLAMIC REPUBLIC OF

## Abstract

Apple (*Malus domestica Borkh*.) is one of the most economically important fruit crops worldwide; however, its production is severely constrained by major diseases, including fire blight, powdery mildew, and apple scab. Breeding disease-resistant cultivars represents a sustainable alternative to chemical control, particularly through the effective utilization of local germplasm resources from Central Asia. This study aimed to evaluate the presence and distribution of resistance-associated alleles in local apple varieties from Uzbekistan using polymorphic DNA markers. A collection of local apple accessions was screened to identify markers linked to resistance against fire blight, powdery mildew, and apple scab. The analysis revealed substantial genetic variation in resistance gene combinations among the studied varieties. The fire blight-associated marker AE10-375 was detected in 79.8% of the accessions. For powdery mildew resistance, 75.2% of the varieties carried resistance alleles corresponding to both Pl1 and Pl2 genes. Screening for apple scab resistance demonstrated that Vfa2*, Vfa1*, and Rvi6 were the most prevalent genes, with Vfa2 detected in 95.4% of the accessions. Regional analysis indicated that accessions from Karakalpakstan exhibited the highest proportion of genotypes harboring markers associated with resistance to multiple diseases. Six local varieties—Atlas olma, Turkish, Xuboni, Krasniy jeleznyak, Shoyi olma, and Besh barmoq—were identified as carrying resistance-associated markers for all three diseases. These findings demonstrate that local apple germplasm from Uzbekistan represents a valuable genetic resource for resistance to economically important diseases. The identified genotypes provide promising donor material for breeding programs aimed at developing cultivars with durable, broad-spectrum resistance while reducing reliance on chemical control strategies.

## Introduction

The modern apple was domesticated through trade along the Silk Road from its wild crabapple progenitors in the Tien Shan Mountains of Central Asia [1]. The genetic diversity found in this region encompasses valuable germplasm that can be exploited in breeding programs targeting agronomically important traits and disease resistance [2]. Through domestication, alleles conferring disease resistance and response to abiotic stress have been lost because their linkage with unfavorable alleles affects horticultural traits such as fruit size. Climate change and a growing global population heighten the urgency of improving existing crop species or developing new cultivars with disease resistance and environmental resilience.

Severe diseases in apples are caused by both bacterial and fungal pathogens. Among them, the most common diseases are fire blight – FB (*Erwinia amylovora*), powdery mildew – PM (*Podosphaera leucotricha*), and apple scab – AS *(Venturia inaequalis)* [3–5]. Orchards affected by fungal diseases such as AS and PM require intensive plant protection management, with up to 25 fungicide applications required per vegetation period in some regions. The large input of fungicides affects both the environment and human health and increases the price of apple fruits. Furthermore, overuse of fungicides imposes selective pressure on pathogen populations, promoting the development of resistance to chemical agents [6].

Host genetic resistance is a sustainable alternative management strategy to reduce fungicide inputs [7]. Many commercial cultivars grown widely today are susceptible to the major pathogens of apple. Early studies identified wild apple species, including *M. robusta, M. sublobata, M. atrosanguinea, M. prunifolia*, and *M. fusca*, are carriers of genetic resistance to various diseases [8]. Despite these findings, there have been limited efforts to incorporate these traits into new cultivars. This is primarily due to the long breeding cycle of apple and the multiple rounds of breeding necessary to reduce the linkage drag of undesirable traits such as poor fruit quality. The goal of many apple breeding programs is to combine important horticultural traits with disease resistance [9]. To accelerate resistance breeding, molecular markers associated with resistance to AS, PM, and FB have been developed and are increasingly deployed in marker-assisted selection (MAS) programmes [4,10–17].

The importance of local and regional germplasm as a source of resistance alleles is increasingly recognized [12]. Local landraces, shaped by centuries of farmer-driven selection under region-specific disease pressure, represent a particularly relevant source of resistance alleles for breeding programs targeting agroecological conditions similar to those in which they evolved. Unlike exotic wild relatives, local varieties may carry resistance combined with desirable horticultural traits, thereby reducing the challenge of linkage drag in breeding crosses.

Uzbekistan, located in Central Asia, has a long history of apple cultivation and breeding. Its close geographic proximity to the primary center of origin of the modern apple suggests the presence of substantial varietal diversity. In recent years, an increased incidence of FB and PM has been reported in the country, posing a growing threat to apple production [18,19]. Despite the substantial diversity of Uzbek apple germplasm and its proximity to the primary centre of origin of domesticated apple, no comprehensive molecular evaluation of resistance to major pathogens has been conducted in this gene pool. Previous studies have focused on European and North American cultivar collections; local Central Asian landraces remain largely unexplored as sources of resistance alleles for contemporary breeding. The present study fills this gap by applying validated DNA markers to a broad collection of local accessions from three geographically distinct regions of Uzbekistan.

The present study aimed to: (i) screen 109 local Uzbek apple accessions for the presence of DNA markers associated with resistance to FB, PM, and AS; (ii) evaluate phenotypic disease resistance under natural field conditions across three geographically distinct regions; and (iii) identify multi-disease-resistant accessions with potential as donor parents in resistance breeding programs. These results provide valuable information for breeders and growers and establish a foundation for further genetic and breeding studies on apple varieties originating from Central Asia.

## Materials and methods

### Plant material

Young leaves were collected from 109 local apple (*Malus domestica*, *Malus sieversii*, and *Malus niedzwetzkyana*) accessions from three geographically distinct regions of Uzbekistan: the northwestern provinces (Karakalpakstan, n = 33; and Khorezm Province, n = 41) and the southern Surxondaryo Province (n = 35). Sampling was designed to capture geographic and varietal diversity across these three distinct agro-ecological zones. Farms were selected in consultation with regional agricultural specialists from the M. Mirzaev Scientific Research Institute of Horticulture, Viticulture, and Winemaking; the Scientific Research Institute of Forestry; and the Uzbek Scientific Research Institute of Plant Growing, to represent historically important apple-growing districts within each province. Within each farm, one tree per variety was sampled as a minimum; where multiple trees of the same variety were present, samples were collected from each tree to account for potential within-variety genetic diversity arising from vegetative propagation over generations.

Although accessions shared varietal names, they represent farmer-maintained local landraces propagated over generations, and therefore are not assumed to be genetically identical. Phenotypic characterization and cultivar identification were conducted collaboratively with specialists from the three institutes listed above. All samples were collected with the consent of farm owners and in full compliance with the national regulations of the Republic of Uzbekistan regarding the use of plant genetic resources. Ethics approval and consent to participate: Not applicable. The geographic location of each collection site was recorded using GPS. Each variety was assigned a sample code based on geographic origin, where the first letter(s) correspond to the region; detailed information on variety name, farm owner, and district is provided in Supplementary Table 1. Leaf samples were placed into individual Ziplock bags containing silica gel and transported to the laboratory for subsequent DNA isolation and molecular analysis.

No voucher specimens were deposited, as all samples originated from clearly identified cultivated apple varieties maintained in traditional orchards.

### Evaluation of disease symptoms

The evaluation of apple trees was performed under natural (uncontrolled) conditions on private farms over two growing seasons (2022 and 2023). Disease severity was assessed at two time points per season (early and late in the infection period), and scores were averaged to obtain a representative resistance index for each tree. Farms with no visible disease incidence in either year were excluded from phenotypic analysis, although molecular data were retained for all accessions. Assessments of disease severity were made on each tree, and values of leaf and fruit incidence from the quadrants were averaged to obtain the percentage of diseased leaves and fruit per tree [20].

Disease severity for FB, PM, AS was evaluated using a standardized five-point ordinal scale (0–4), adapted from methodologies established by van der Zwet [21], Kim [22], and Rexhepi [23]. This scale facilitates consistent phenotypic classification of plant accessions into resistance categories: highly resistant (score 4), moderately resistant (score 3), weakly resistant (score 2), and susceptible (score 1 and 0). Disease-specific criteria were defined as follows:

- Fire blight: Score 4 = asymptomatic; score 3 = 1–12% of canopy exhibiting blight symptoms; score 2 = 13–50% canopy affected; score 1 = 51–75% canopy affected; score 0 = > 75% canopy affected.
- Powdery mildew: Score 4 = no visible symptoms; score 3 = 1–5% of leaf area colonized; score 2 = 5.1–20% leaf area affected; score 1 = 20.1–40% leaf area affected; score 0 = > 40.1% leaf area affected.
- Apple scab: Score 4 = symptomless foliage and fruit; score 3 = 1–10% of leaf and fruit surface exhibiting lesions; score 2 = 10.1–25% surface affected; score 1 = 25.1–50% surface affected; score 0 = > 50.1% surface affected.

### Molecular analysis

The present study does not aim to develop novel markers, but rather to apply validated resistance-associated markers to an underexplored Uzbek local apple germplasm. Genomic DNA from each variety was extracted using the PureLink® Plant Total DNA Purification Kit (Thermo Fisher Scientific, Waltham, MA, USA) according to the manufacturer’s instructions. DNA quality was assessed spectrophotometrically on a BioSpec-nano (Shimadzu Corporation, Kyoto, Japan): only samples with an A260/A280 ratio of 1.8–2.0 and an A260/A230 ratio > 1.5 were used for PCR amplification. All DNA samples were diluted to 20–30 ng/µl, visualized on a 0.8% agarose gel, and stored at −20°C for further analysis. PCR was performed by using primer pairs specific to resistance markers to FB: AE10–375, CH-F7-Fb1, and GE-8019 [14]; PM: AT20–450 and Pm739 [24]; and AS: VfC, AM19, and AL07 [10,11]. All primer sequences, annealing temperatures, and expected amplicon sizes are listed in Table 1.

**Table 1:** List of the molecular markers for AS, FB and PM resistance loci/genes along with their primer sequences, annealing temperatures and amplicon sizes.

№	Marker name	Resistance gene / QTL	Primer pairs	Та* (°C)	PCR band size** (b.p.)	Reference
**Fire blight**						
1	AE10–375	*FBF7 QTL (LG7)*	F 5’-ctgaagcgcacgttctcc-3’ R 5’-ctgaagcgcatcatttctgatag-3’	60	**375** / -	[14]
2	CH-F7-Fb1	*FBF7 QTL (LG7)*	F 5’-agccagatcacatgttttcatc-3’ F 5’-acaacggccaccagtttatc-3’	60	**210** / 174	
3	GE-8019	*FBF7 QTL (LG7)*	F 5’-ttgagaccgattttcgtgtg-3’ R 5’-tctctcccagagcttcattg-3’	60	**397** / -	
**Powdery mildew**						
4	AT20–450	*Pl-1*	F 5’-atcagccccacatgaatctcatacc-3’ R 5’-acatcagccctcaaagatgagaagt-3’	62	**450** / 500	[24]
5	Pm739	*Pl-2*	F 5’-aacgaagatcaagaaacatgg-3’ R 5’-tgccgtcaatggaacacc-3’	53	**269** / -	
**Scab**						
6	VfC	*Vfa1 (HcrVf1), Vfa2 (HcrVf2), Vfa4*	F 5’-ggtttccaaagtccaattcc-3’ R 5’-cgttagcattttgagttgac-3’	58	**286 /** 484 / 646	[11]
7	AM19	*Rvi6*	F 5’-cgtagaacggaatttgacagtg-3’ R 5’-gacaaagggcttaagtgctcc-3’	60	**526 / -**	[10]
8	AL07	*Rvi6*	F-5’-tgg aag aga gat cca gaa agt g-3’ R-5’-cat ccc tcc aca aat gcc-3’	60	**466/724**	[10]

*Ta: annealing temperature. **PCR band size: fragment sizes corresponding to resistant and susceptible alleles as reported in the original publications; “–” indicates absence of amplification.

PCR amplification was performed using 20 ng of genomic DNA in 10 µl volumes containing 10 mM Tris-HCl (pH 8.3), 50 mM KCl, 1.5 mM MgCl2, 100 µM each dNTP, 200 nM each primer and 0.6 U Taq polymerase. PCR amplification was carried out on a Veriti™ Thermal Cycler (Thermo Fisher Scientific) using an initial denaturation step at 95°C for 5 min, followed by 35 cycles of 40 s at 94°C, 1 min at annealing temperatures based on the primers used for each marker (Table 1), 1 min at 72°C, and a final extension at 72°C for 10 min. All PCR amplifications were performed in duplicate; discrepant results were resolved by a third independent reaction. Negative (water) controls were included in every PCR run.

Amplified products were separated on 2.5% agarose gels in 0.5 × TBE, stained with ethidium bromide, and photographed under UV illumination. Fragment sizes were scored against a 100 bp DNA ladder (NEB). For dominant markers, a band was scored as present (+) if a fragment of the expected size (±5 bp) was observed; absence of any band was scored as (−). For codominant markers (CH-F7-Fb1, VfC, AL07), individual alleles were identified based on their diagnostic fragment sizes. Scoring was performed independently by two researchers; discrepancies were resolved by a third independent assessment.

### Statistical analysis

PCR band presence/absence data were coded as binary (1/0) for each variety per marker. Statistical analysis was performed in GenAIEx 6.5 [25,26]. Pairwise Nei’s genetic identity between populations was calculated across markers associated with each disease. Genetic relationships between accessions were analyzed by cluster analysis using the neighbor-joining (NJ) method; dendrograms were constructed using MEGA12 software [27]. Evolutionary distances were computed using the p-distance method. Principal component analysis (PCA) was performed to explore genetic structure using the dplyr, ggplot2, ggrepel, and factoextra libraries in R Studio (version 4.4.2). Spearman rank correlation analysis was performed between resistance index scores and marker presence to assess marker-phenotype associations. Chi-square tests were used to test for significant differences in marker allele frequencies across geographic regions. Statistical significance was set at α = 0.05.

## Results

### Fire blight resistance markers

The dominant SCAR markers AE10–375 and GE-8019 associated with alleles linked to the *FBF7* fire blight resistance QTL [14] produced clear single bands of 375 bp and 397 bp in 79.81% and 11.0% of samples, respectively (Table 2). Among these accessions, 77 amplified the AE10–375 locus only, whereas 2 amplified GE-8019 alone. Both loci were amplified in 10 accessions, whereas 20 accessions failed to amplify either marker. The *FBF7*-resistant allele of GE-8019 was absent in Khorezm accessions, and was distributed in Surkhandaryo and Karakalpak regions in 16% and 12% of accessions, respectively. The dominant marker AE10–375 was found in 92% of Surkhandaryo and 88% of Khorezm samples (Table 2). Overall, 83.48% of the Uzbek apple accessions contained at least one *FBF7*-associated locus. The codominant STS marker CH-F7-Fb1 produced amplification products of 174 and 210 bp, the latter associated with resistance, and was present in 91 accessions. Spearman rank correlation between fire blight resistance index and marker presence was significant (rs = 0.51, p < 0.001). Chi-square analysis revealed statistically significant differences in AE10–375 frequency across regions (χ2 = 18.4, df = 2, p < 0.001).

**Table 2:** Molecular marker genotypes of 109 local apple varieties showing the presence (+) or absence (–) of resistance-associated alleles for *E. amylovora*, *P. leucotricha*, and *V. inaequalis.*

		Marker genotypes for resistance to fire blight (*E.amylovora*)				Marker genotypes for resistance to powdery mildew (*P.leucotricha*)			Marker genotypes for resistance to scab (*V.inaequalis*)						
		GE-8019	AE10–375	CH-F7Fb1 (210 bp)	Resistance Index^b^	AT20–450	Pm739	Resitance Index	VfC			AM19	AL07		
№^a^	Variety					*Pl1*	*Pl2*		*Vfa1* (646 bp)	*Vfa2* (484 bp)	*Vfa4* (286 bp)	*Vf* (526 bp)	*Vf* (466 bp)	*Vf* (724 bp)	Resistance Index
SS01	Qirmizak	–	+	–	2	+	+	3	–	+	–	–	–	+	3
SS02	Renet Semerenko	–	+	–	2	+	+	4	–	+	–	–	–	+	3
SS03	Atlas olma	–	+	+	4	+	+	4	–	+	–	+	–	+	4
SS04	Turkish	–	+	+	4	+	+	4	–	+	–	+	–	+	4
SS05	Atlas kechki	+	+	–	4	–	+	3	–	+	–	–	–	+	3
SS06	Renet Simarenko	–	+	+	3	–	+	3	–	+	–	–	–	+	3
SS07	Qirmizak	–	+	+	4	+	+	4	–	+	–	–	–	+	3
SS08	Starkrimson	–	+	+	4	+	+	4	–	+	–	–	–	+	3
SS09	*Malus niedzwetzkyana*	–	+	+	4	–	–	2	–	+	–	–	–	+	3
SS10	Xuboni	–	+	+	4	+	+	4	–	+	–	+	–	+	4
SS11	Sariq olma	+	+	+	4	+	–	3	–	+	–	–	–	+	3
SD01	Mayskiy	–	–	–	1	–	–	1	+	+	–	–	–	+	2
SD02	Xuboni	–	+	+	4	+	–	3	+	+	–	–	–	+	2
SD03	Mayskiy	–	+	+	4	+	–	3	–	+	–	–	–	+	3
SD04	Xuboni	–	+	–	2	+	+	4	–	+	–	–	–	+	3
SD05	Atlas olma	–	+	–	2	+	+	4	–	+	–	–	–	+	3
SD06	Mayskiy Qizil	–	+	–	2	+	–	3	+	+	–	–	–	+	2
SD07	Eshak olma	–	–	–	1	–	+	3	+	+	–	–	–	+	2
SD08	Qizil kechki olma	–	+	+	4	+	–	3	–	+	–	–	–	+	3
SD09	Oq olma	–	–	–	2	–	+	3	–	+	–	–	–	+	3
KH01	Besh barmoq	–	+	+	3	+	+	4	–	+	–	–	–	+	3
KH02	Renet Semerenko	+	–	–	2	+	+	4	–	+	–	+	–	+	4
KH03	Samarqandskiy ranniy	–	+	+	3	+	+	4	–	+	–	+	–	+	4
KH04	Starkrimson	–	+	–	2	+	+	4	–	+	–	–	–	+	3
KH05	Golden delicious	–	+	–	2	+	+	4	–	+	–	–	–	+	3
KH06	Jonathan	–	+	+	3	–	+	3	–	+	–	–	–	+	3
KH07	Pervenets Samarqanda	–	+	+	3	+	+	4	–	+	–	–	–	+	3
KH08	Besh yulduz	–	+	+	3	–	+	3	–	+	–	–	–	+	3
KH09	Besh barmoq	–	+	–	2	–	+	3	–	+	–	–	–	+	3
KH10	Grimes Golden	+	+	–	4	–	+	3	–	+	–	+	–	+	4
KH11	Qand olma kishgi	–	+	+	3	+	+	4	+	+	–	+	–	+	3
KH12	Mayskiy	–	+	+	3	+	+	4	–	+	–	+	–	+	4
KH13	Red delicious	–	+	+	3	+	+	4	–	+	–	+	–	+	4
KH14	Qizil olma urtagi	–	+	–	2	+	+	4	–	+	–	–	–	+	3
KH15	Qizil olma	–	+	+	3	–	+	3	–	+	–	–	–	+	3
KH16	Shoyi olma	–	+	+	3	+	+	4	–	+	–	+	–	+	4
KH17	Tosh olma	–	+	–	2	–	+	3	+	+	–	–	–	+	2
KH18	Qand olma	–	+	+	3	–	+	3	+	+	–	–	–	+	2
KH19	Krasniy jeleznyak	+	+	–	4	–	+	3	+	+	–	+	–	+	3
XS01	Besh barmoq	–	+	–	2	+	+	4	–	+	–	+	–	+	4
XS02	Grimes Golden	–	+	+	2	+	–	3	+	–	–	+	–	+	2
XS03	So’x go’zali	–	+	+	3	+	+	4	–	+	–	–	–	+	3
XS04	R.Semerenko	+	+	–	4	+	+	4	–	+	–	–	–	+	3
XS05	Qizil sho’r olma	–	+	–	2	+	+	4	–	+	–	–	–	+	3
XS06	Golden delicious	–	+	+	3	+	+	4	+	–	–	–	–	+	2
XS07	Yozgi olma	–	+	+	3	–	+	3	–	+	–	–	–	+	3
XS08	Krasniy jeleznyak	+	+	–	4	+	+	4	+	+	–	–	–	+	2
XS09	Mayskiy	–	+	–	2	+	+	4	–	+	–	–	–	+	3
XS10	Qizil olma	+	+	–	4	+	+	4	–	+	–	–	–	+	3
XS11	Xazaraspskiy letniy	–	+	+	4	+	+	4	–	+	–	–	–	+	3
XS12	Qand olma kishgi	–	+	–	2	+	+	4	–	+	–	–	–	+	3
XS13	Qand olma	+	+	–	4	+	+	4	+	+	–	–	–	+	2
XS14	Muz olma	–	+	–	2	+	+	4	+	–	–	–	–	+	1
XS15	Xivinskiy beshbaroq	–	+	+	4	+	+	4	–	+	–	–	–	+	3
SSh01	Renet Semerenko	–	–	+	1	+	+	4	–	+	–	–	–	+	3
SSh02	Oq olma	–	–	–	1	+	+	4	+	+	–	+	–	+	3
SSh03	Besh yulduz	–	–	–	1	–	+	3	+	+	–	+	–	+	3
SSh04	Qizil olma	–	–	–	1	–	+	3	+	+	–	+	–	+	3
SSh05	Besh yulduz	–	–	–	1	–	+	3	+	+	–	+	–	+	3
SSh06	Renet Semerenko	–	–	–	1	–	+	3	+	+	–	+	–	+	3
SSh07	Oq olma	–	–	–	1	+	+	4	+	+	–	+	–	+	3
SSh08	Besh yulduz	–	+	+	4	+	+	4	+	+	–	–	–	+	2
KB01	Krasniy jeleznyak	–	+	+	4	+	+	4	–	+	–	+	–	+	4
KB02	Jonathan	–	+	+	4	+	+	4	–	+	–	–	–	+	3
KB03	Yozgi olma	–	–	–	1	+	+	4	–	+	–	–	–	+	3
KB04	Starkrimson	–	+	–	2	+	+	4	+	+	–	–	–	+	2
KB05	Xazaraspskiy letniy	–	–	–	1	+	+	4	–	+	–	–	–	+	3
KB06	Xazaraspskiy zimniy	–	–	–	1	+	+	4	+	+	–	–	–	+	2
KB07	Shoyi olma	+	–	–	2	+	+	4	+	–	–	–	–	+	1
KB08	Besh yulduz	–	+	–	2	+	+	4	+	–	–	–	–	+	2
KB09	Golden delicious	–	+	–	2	+	+	4	+	+	–	–	–	+	2
KB10	*Malus niedzwetzkyana*	–	+	+	4	+	+	4	+	+	–	–	–	+	2
KB11	Rozemary white	–	+	+	4	+	+	4	–	+	–	–	–	+	3
KB12	Besh barmoq	–	+	–	2	+	+	4	+	+	–	–	–	+	2
KB13	Yanar	–	+	+	4	+	+	4	+	+	–	–	–	+	2
KB14	Qizil olma (Red apple)	–	+	+	4	+	+	4	–	+	–	–	–	+	3
SKO01	Qizil olma (Red apple)	–	+	–	2	+	+	4	+	+	–	–	–	+	2
SKO02	Qizil olma (Red apple)	–	+	–	2	+	+	4	+	+	–	+	–	+	3
SKO03	Qizil olma (Red apple)	–	+	–	2	+	+	4	–	+	–	+	–	+	4
SKO04	Qizil olma (Red apple)	–	+	–	2	+	+	4	–	+	–	+	–	+	4
SKO05	Renet Semerenko	–	+	–	2	+	+	4	+	+	–	+	–	+	3
SKO06	Oq olma	–	+	–	2	+	+	4	+	+	–	–	–	+	2
SKO07	Besh yulduz	–	+	–	2	+	+	4	+	+	–	+	–	+	3
SKO08	Oq olma	–	+	–	2	+	+	4	–	+	–	+	–	+	4
SKO09	Oq olma	–	–	–	1	+	+	4	–	+	–	+	–	+	4
SKO10	Oq olma	–	+	–	2	+	+	4	–	+	–	+	–	+	4
SKO11	Besh yulduz	–	+	–	2	+	+	4	+	+	–	+	–	+	3
SKO12	Oq olma	–	+	–	2	+	+	4	+	+	–	+	–	+	3
SKb01	Besh yulduz	–	+	+	4	+	+	4	+	+	–	+	–	+	3
Skb02	Atlas	–	+	–	2	+	+	4	+	+	–	–	–	+	2
SKb03	Qizil olma	–	–	+	2	+	–	3	–	+	–	–	–	+	3
SKb04	Oq olma	–	+	–	2	+	+	4	–	+	–	–	–	+	3
SKb05	Qizil olma	–	–	–	1	+	+	4	–	+	–	–	–	+	3
SKb06	Qizil olma	–	+	–	2	+	+	4	–	+	–	–	–	+	3
SKb07	Oq olma	–	+	–	2	+	+	4	–	+	–	–	–	+	3
SKb08	Renet Semerenko	–	–	–	1	+	+	4	–	+	–	+	–	+	4
SKb09	Qizil olma	–	–	–	1	+	+	4	–	+	–	+	–	+	4
SKb10	Qizil olma	–	+	–	1	+	+	4	–	+	–	–	–	+	3
XK01	Karvak qutir olma	–	+	–	1	+	+	4	–	+	–	–	–	+	3
XK02	Avgustovskiy	–	–	–	1	–	–	1	–	+	–	–	–	+	4
XK03	Ruxi janon	–	+	–	1	+	+	4	–	+	–	–	–	+	4
XK04	Qand olma	–	+	–	1	+	+	4	–	+	–	–	–	+	3
XK05	Karvak olma	+	+	–	2	+	+	4	–	+	–	–	–	+	3
XK06	Qutir qand olma	–	+	+	3	+	+	4	–	+	–	–	–	+	3
XK07	Jeleznyak	–	–	–	1	+	+	4	+	+	–	+	–	+	3
XK08	Muz olma	–	+	–	2	+	+	4	–	+	–	+	–	+	3
XK09	Renet Semerenko	–	+	+	3	+	+	4	+	+	–	–	–	+	4
XK10	Besh barmoq	+	+	+	4	+	+	4	–	+	–	+	–	+	2
XK11	Red delicious	–	+	–	2	+	+	4	+	+	–	+	–	+	4

a-The letters in the accession number were assigned depending on their location in the geographical region of Uzbekistan. S -Surkhandarya, K- Karakalpakstan, X – Khorezm. b-The resistance index was assigned depending on the degree of pathogen infestation; the disease resistance indices were as follows: 1-susceptible, 2-low resistance, 3-moderate resistance, and 4-high resistance.

### Apple scab resistance markers

The dominant marker AM19 and the codominant AL07 marker, both associated with *Rvi6* [formerly *HcrVf2*] apple scab resistance, were amplified in 33.94% and 100% of accessions, respectively. The codominant AL07 marker amplifies two alleles: a 466 bp fragment associated with resistance and a 724 bp susceptibility-associated fragment. The 724 bp allele was found in 100% of accessions, whereas the 466 bp resistance allele was not amplified in any accession. The VfC marker detected two loci, *Vfa1* (646 bp) and *Vfa2* (484 bp); no *Vfa4* (286 bp)-specific amplification was identified. The *Vfa2* allele was identified in 95.41% of accessions, whereas *Vfa1* was present in 36.69% of accessions. The distribution of loci was uneven among populations; *Vfa1* was found primarily in Karakalpak accessions (9.1%), followed by Surkhandaryo (4%), and was not detected in the Khorezm region (Table 2). Pairwise Nei’s genetic identity via VfC loci revealed the highest similarity between Karakalpak and Surkhandaryo accessions (97.5%), and the lowest similarity between Khorezm and Surkhandaryo accessions (84.3%) (Table 3).

**Table 3:** Pairwise Nei’s genetic identity among apple accessions from different geographic regions on the basis of loci associated with pathogen resistance.

	Surkhandarya			Karakalpak		
	Fire blight (*E.amylovora*)	Scab (*V.inaequalis*)	Powdery mildew (*P.leucotricha*)	Fire blight (*E.amylovora*)	Scab (*V.inaequalis*)	Powdery mildew (*P.leucotricha*)
**Surkhandarya**	1	1	1	–	–	–
**Karakalpak**	0.997	0.975	0.995	1	1	1
**Khorezm**	0.967	0.843	0.967	0.971	0.865	0.988

### Powdery mildew resistance markers

The *Pl-1* resistance allele was found in 71.3% of varieties in the homozygous state and in 14.8% in the heterozygous state. The Khorezm region contained the most accessions with the *Pl-1* allele (90.4%), followed by Karakalpak (78.8%) and Surkhandaryo (72.4%). The dominant Pm739 marker for *Pl-2* resulted in amplification of a single 269 bp product in 33 Karakalpak, 24 Khorezm, and 42 Surkhandaryo accessions (Table 2). Pairwise Nei’s genetic identity was highest between Surkhandaryo and Karakalpak accessions (99.5%); Khorezm and Surkhandaryo presented the lowest identity of 96.7% (Table 3). Spearman correlation between PM resistance index and combined *Pl-1/Pl-2* marker presence was significant (rs = 0.44, p < 0.001). Chi-square analysis confirmed significant regional differences in *Pl-1/Pl-2* allele distribution (χ2 = 12.7, df = 2, p = 0.002).

### Phylogenetic analysis and cluster structure

**Fig 1:**
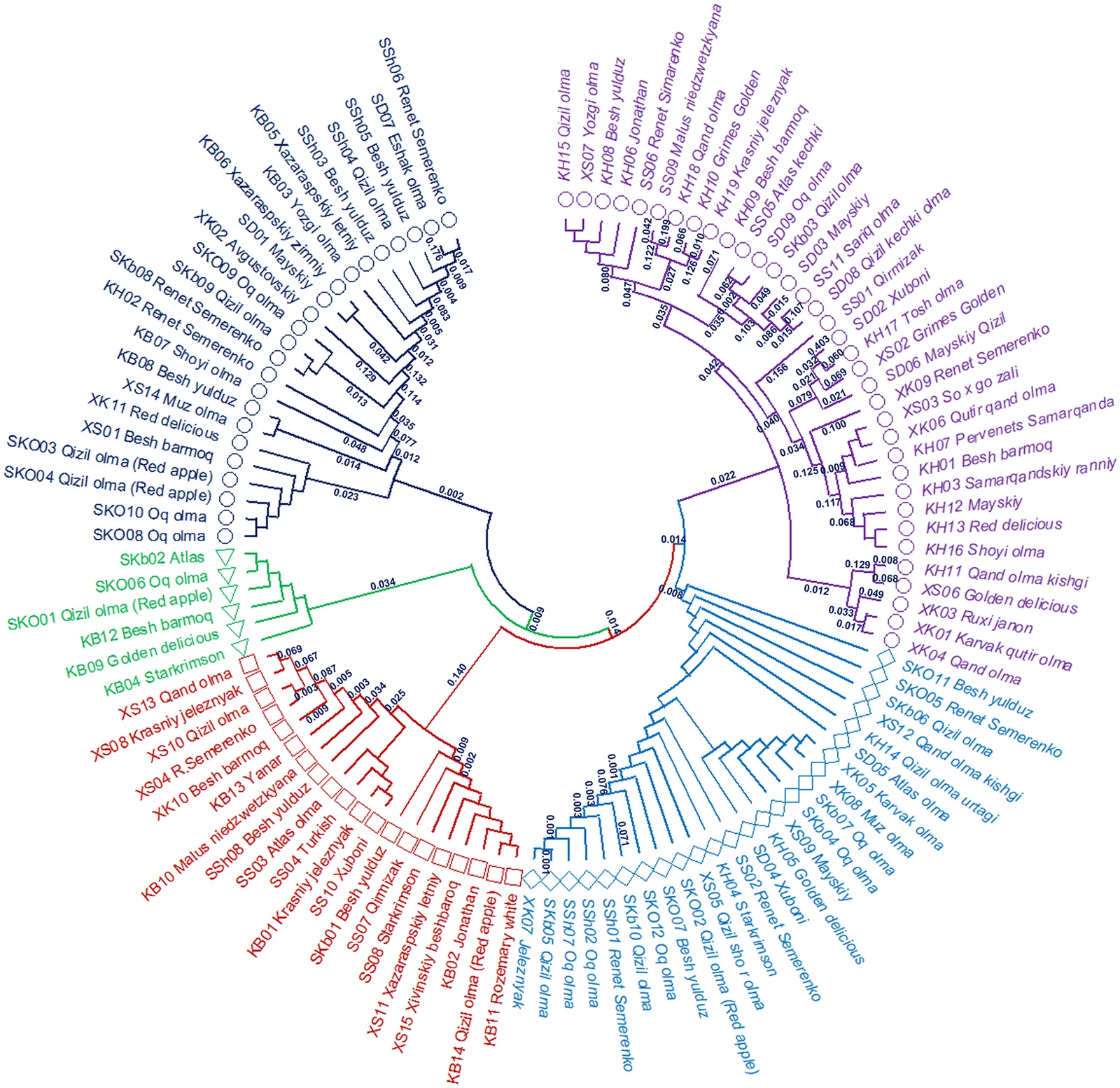
Neighbor-joining phylogenetic tree of 109 Uzbek apple cultivars based on resistance marker data from all eight markers (AE10-375, CH-F7-Fb1, GE-8019, AT20-450, Pm739, VfC, AM19, AL07). Evolutionary distances were computed using the p-distance method in MEGA12. Five biologically distinct clusters are identified: (i) Blue cluster — accessions carrying AE10-375 without *Vfa1*, with a single PM resistance allele, corresponding to moderate PM resistance and low-to-moderate FB and AS resistance; (ii) Green cluster — accessions lacking both AE10-375 and GE-8019 but carrying both *Pl-1* and *Pl-2*, corresponding to high PM resistance combined with low FB resistance; (iii) Red cluster — accessions with multiple FB marker combinations (AE10-375 + CH-F7-Fb1 210 bp or GE-8019 + CH-F7-Fb1) together with both PM alleles, corresponding to moderate–high FB and PM resistance; (iv) Light blue cluster — accessions with *Vfa2* and both PM alleles but without AE10-375, corresponding to strong PM and AS resistance with reduced fire blight protection; (v) Purple cluster — accessions distributed across all three regions sharing an intermediate marker genotype (single-allele FB markers, *Vfa2* only, one PM allele), corresponding to broad but moderate resistance across all three diseases.

To visualize genetic relationships among accessions based on their combined resistance marker profiles, a neighbor-joining (NJ) phylogenetic tree was constructed for all 109 accessions using eight markers simultaneously (Fig 1). The analysis grouped accessions into five biologically meaningful clusters that reflect distinct combinations of resistance alleles across the three diseases.

The blue cluster (predominantly Surkhandaryo accessions) comprised varieties carrying the AE10−375 fire blight marker but lacking the *Vfa1* scab allele and carrying only one PM resistance allele. This marker profile corresponds to the phenotypic pattern of moderate PM resistance combined with low-to-moderate resistance to FB and AS observed in this region. The green cluster (mixed regional origin) contained accessions characterized by the absence of both FB-associated SCAR markers (AE10−375 and GE-8019) combined with strong *Pl-1* and *Pl-2* PM allele presence, explaining the phenotypic pattern of high PM resistance despite low fire blight resistance. The co-localization of *Pl-1* and *Pl-2* on chromosome 12 likely maintains their coupling in this cluster [28].

The red cluster contained accessions with the most complete fire blight marker combinations (AE10–375 + CH-F7-Fb1 210 bp, or GE-8019 + CH-F7-Fb1), reflecting high-to-moderate FB resistance scores, combined with both *Pl-1* and *Pl-2* PM alleles. The simultaneous presence of multiple FB and PM resistance alleles in this cluster explains the consistently elevated resistance indices for both diseases. The light blue cluster comprised accessions with AE10–375 absent but carrying *Vfa2* and both PM alleles, corresponding to the phenotype of strong PM and AS resistance with reduced fire blight protection. Finally, the purple cluster contained accessions distributed across all three regions sharing an intermediate marker genotype: single-allele FB markers, *Vfa2* only (no *Vfa1*), and at least one PM allele — a combination corresponding to broad but moderate resistance across all three diseases.

Overall, clustering revealed distinct molecular differentiation between regional apple varieties consistent with independent, region-specific selection histories. Karakalpak accessions were overrepresented in the red and light blue clusters (higher combined resistance), while Surkhandaryo accessions were more evenly distributed across all clusters, reflecting the broader phenotypic diversity observed in that region (Fig 1).

### Geographic distribution of phenotypic resistance

**Fig 2:**
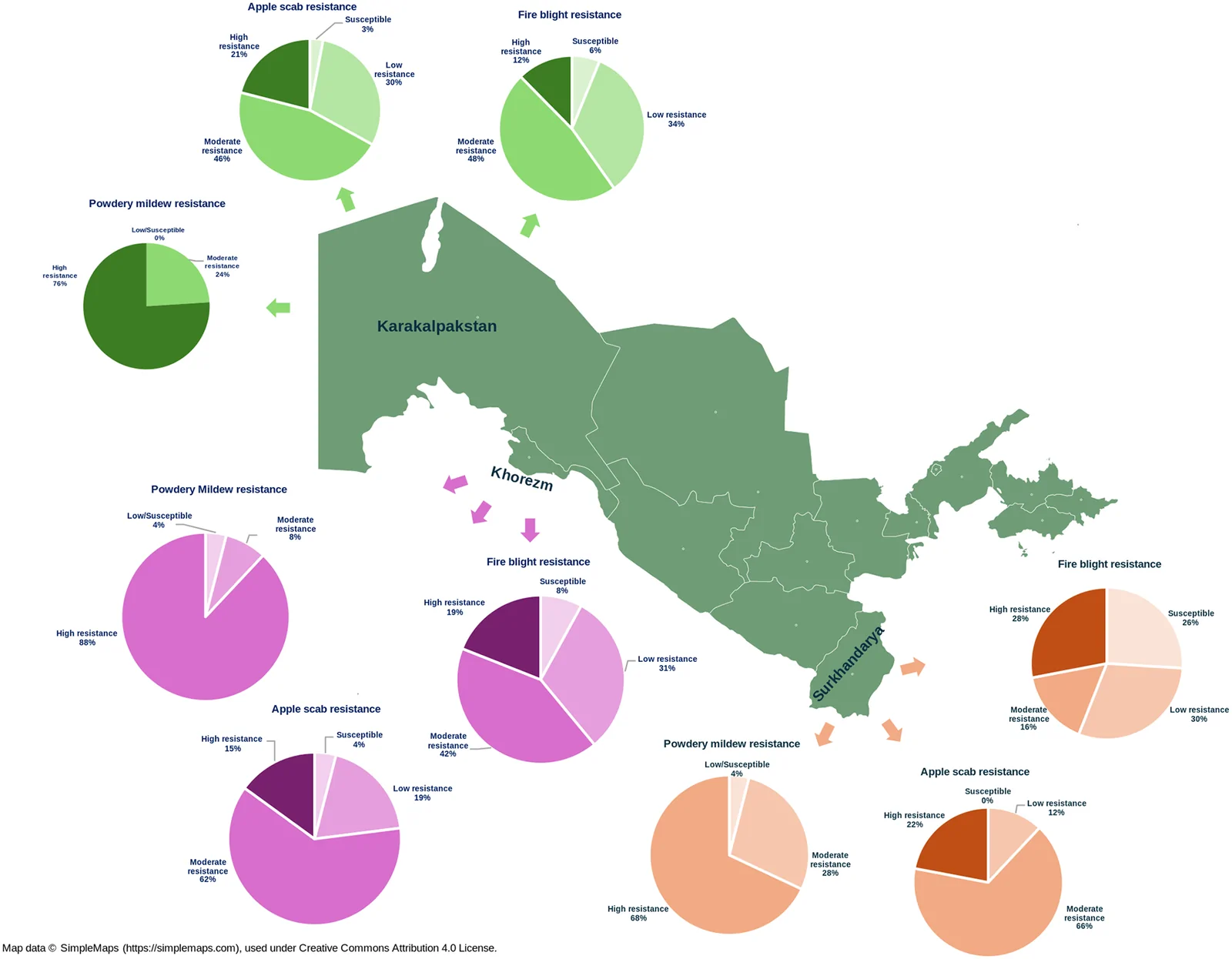
Geographic distribution of apple varieties and their phenotypic resistance to major diseases across sampling regions of Uzbekistan. Pie charts show the proportions of varieties exhibiting high, moderate, low resistance, and susceptibility to apple scab (AS), fire blight (FB), and powdery mildew (PM) in Karakalpakstan, Khorezm, and Surkhandarya. Map boundaries generated using publicly available geographic data from SimpleMaps (https://simplemaps.com), used under the Creative Commons Attribution 4.0 License. Resistance levels were determined from phenotypic disease scoring conducted over two growing seasons (2022–2023).

The geographic distribution of apple accessions and their phenotypic resistance ratings are illustrated in Fig 2. A small proportion of accessions in all regions were susceptible to scab and powdery mildew (0–4% across regions). Among Karakalpakstan varieties, at least one PM resistance allele was present in all accessions (Fig 2). High resistance to powdery mildew was detected in old varieties from all three regions: 68% in Surkhandarya, 76% in Karakalpakstan, and 88% in Khorezm — consistent with reports of low PM susceptibility in old German varieties [13].

### Multi-disease resistant accessions

**Fig 3:**
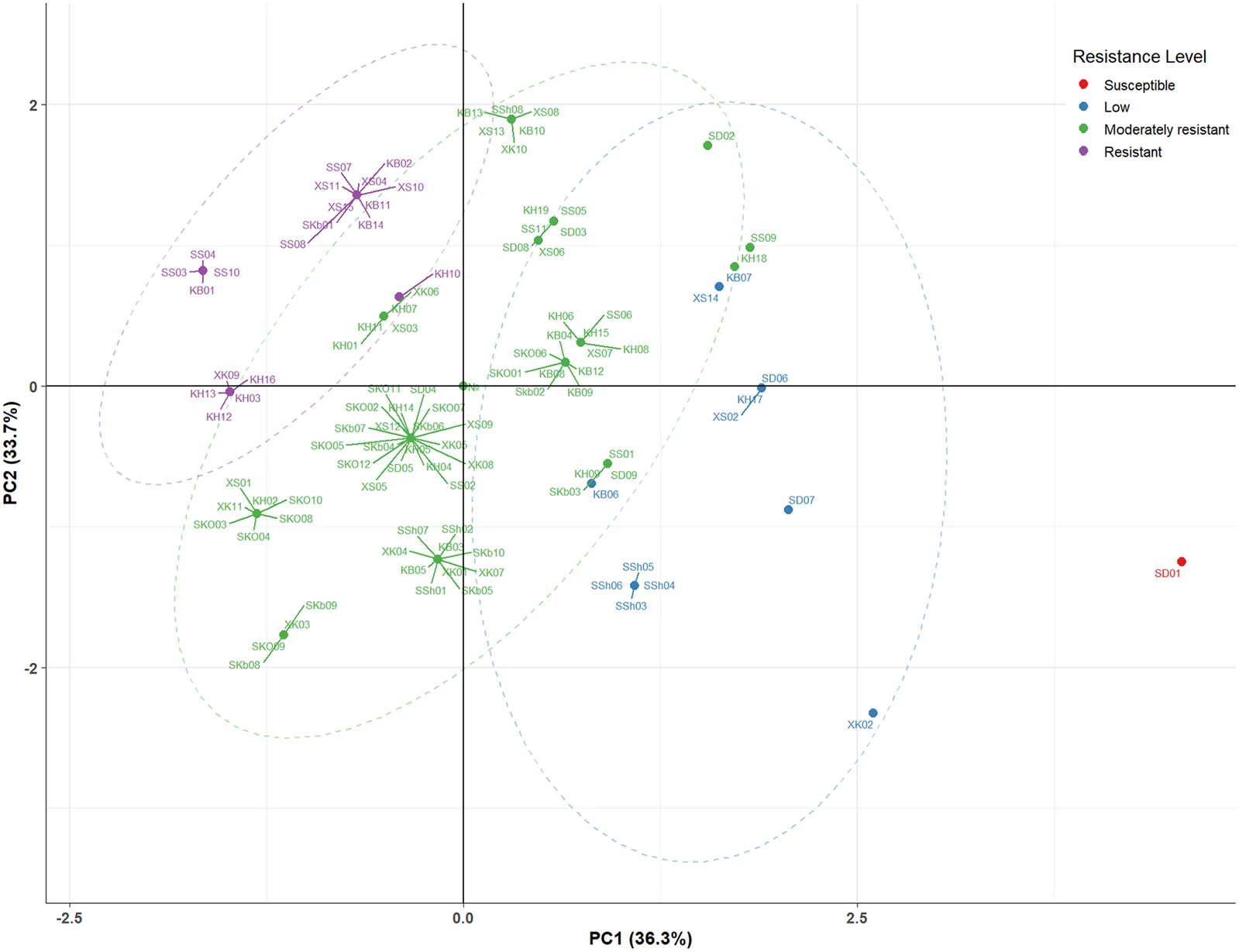
Principal component analysis (PCA) of apple accessions based on resistance indices to fire blight, powdery mildew, and apple scab. Each point represents an individual apple variety positioned along the first two principal components (PC1 = 36.3%; PC2 = 33.7% of total variance). Colors indicate resistance categories: red = susceptible; blue = low resistance; green = moderate resistance; purple = resistant. Ellipses represent 95% confidence intervals for each resistance category. Six multi-disease-resistant accessions identified as carrying resistance-associated markers for all three diseases are labeled.

Principal component analysis visualized the multi-dimensional resistance structure across all accessions (Fig 3). PC1 explained 36.3% and PC2 explained 33.7% of total variance, together capturing most of the inter-accession resistance variation. The PCA revealed clear separation of susceptible and resistant varieties, with most local germplasm falling into low- to moderate-resistance groups. Six local varieties — Atlas olma (SS03), Turkish (SS04), Xuboni (SS10), Krasniy jeleznyak (KB01), Shoyi olma (KH16), and Besh barmoq (XK10) — were identified as carrying resistance-associated markers for all three diseases, making them especially valuable as multi-disease-resistant donor parents in integrated breeding strategies (Fig 3).

## Discussion

This study aimed to characterize local Uzbek apple germplasm using marker-assisted and phenotypic approaches to identify genotypes with combined resistance to major apple diseases. The markers used serve as indicators of the presence of resistance-associated alleles; marker presence indicates potential resistance and should be complemented by phenotypic validation before definitive selection decisions are made.

Most local varieties have been cultivated for many years across multiple regions of Uzbekistan. We found that varieties with the same names but grown in different regions differ in their combination of DNA markers and degree of disease resistance. This suggests that they might not be true to type, even though they continue to be labeled with their original varietal names. It is possible that growers in different regions have favored varieties with greater disease resistance, leading to parallel selective breeding.

The high frequency of resistance-associated marker alleles in the studied germplasm does not always translate to correspondingly high phenotypic resistance scores. This discrepancy reflects the polygenic and quantitative nature of disease resistance in apple. Individual marker loci represent only components of the broader resistance architecture; expression of resistance is modulated by the genetic background, specific pathogen race, and environmental conditions. Epistatic interactions between resistance loci may further contribute to phenotypic variation; complementary gene action between *Rvi6* and additional *Rvi* loci has been reported to enhance resistance beyond that conferred by any single locus [29]. Cases of markers present but low phenotypic resistance may reflect gene silencing, incomplete penetrance, or the presence of pathogen races capable of overcoming specific resistance genes.

The most well characterized resistance gene to apple scab is *Rvi6* (formerly *HcrVf2*), derived from the Japanese crab apple *Malus floribunda 821*. Screening for scab resistance genotypes via the AL07 marker [10] resulted in amplification of a 724 bp susceptibility-associated fragment in all samples; the *Rvi6*-linked 466 bp allele was not detected in any accession. This is striking given that the dominant AM19 was amplified in 33.94% of the varieties and was found to be 0.2 cM to the right of the *Rvi6* locus in the same position as AL07. These data suggest that recombination occurs between the two markers in all Uzbek varieties, which is highly unlikely given the small distance between the markers, or that there is a large nucleotide difference in the region AL07 that is amplified between the *M. floribunda 821* and Uzbek varieties.

The screening of the varietal population for specific paralogs at the *Rvi6* locus was performed via molecular markers to amplify *Vfa1*, *Vfa2*, and *Vfa4* [11]. Malnoy et al. (2008) transformed susceptible ‘Gala’ with each paralog and reported that *Vfa1* and *Vfa2* confer resistance, whereas lines expressing *Vfa4* were more susceptible; later work by Joshi et al. (2011) characterized *Vfa2* as the only functional resistance gene [30,31]. Our work revealed no amplification of the *Vfa4* paralog in any variety. This could indicate that this paralog has diverged in sequence drastically enough in Uzbek varieties to not be detected. *Vfa1* and *Vfa2* were detected in 36.69% and 95.41%, respectively. These results are broadly consistent with findings in Iranian apple germplasm [11]. Varieties rated as susceptible contained the *Vfa1* paralog alone or in combination with *Vfa2,* suggesting that there are races of *Venturia inaequalis* present in Uzbekistan capable of overcoming *Rvi6*-mediated resistance; there are reports of *Rvi6* overcoming races in Europe and North America [32]. Two non-mutually exclusive explanations are proposed for the AL07 discrepancy: (i) sequence divergence in the AL07 primer-binding region in Uzbek varieties relative to the *M. floribunda 821* source from which AL07 was designed; (ii) altered linkage phase between AL07 and AM19 in this germplasm. We recommend future sequencing of the AL07 target region to discriminate between these hypotheses. The genetic assessment of apple varieties for fire blight resistance revealed that only 21.1% of the studied accessions presented high resistance, whereas 35.78% presented low resistance, and 18.35% were susceptible (Table 2). These results indicate that genetic resistance to this disease remains limited among cultivated local apple varieties.

Molecular screening for fire blight resistance via SCAR and SSR markers associated with the AE10–375, GE-8019, and CH-F7-Fb1 loci provides essential insights into the genetic architecture of resistance within the studied germplasm. The dominant SCAR marker AE10–375, which is linked to the *FBF7* locus [14], was detected at a high frequency (79.8%) among the analyzed apple varieties. However, the lack of correlation between molecular genetic results and phenotypic resistance data suggests that this locus alone confers only partial or conditional resistance, which is consistent with previous reports [33,34]. In contrast, the GE-8019 marker was observed at a low frequency (11.0%), indicating its limited distribution in the studied gene pool, which may reflect either its rarity in local germplasmor its potential loss during breeding selection for other agronomic traits. The codominant CH-F7-Fb1 SSR marker, particularly its 210-bp allele, was found to be associated with fire blight resistance and was detected in 37.6% of the studied apple accessions. Notably, only 35.78% of the accessions carried both AE10–375 and CH-F7-Fb1–210, and—contrary to earlier reports suggesting genetic linkage between these two loci [14] —no evidence of cosegregation was observed. The independent segregation of AE10–375 and CH-F7-Fb1 likely reflects recombination events or distinct haplotype structures in the diverse Uzbek germplasm pool with *M.sieversii* and *M. niedzwetzkyana* ancestry. Accessions harboring combinations of multiple fire blight markers consistently presented moderate to high resistance, supporting allele pyramiding as a strategy for durable resistance [16]. Phenotypic resistance data confirmed that apple accessions harboring combinations of AE10–375 and CH-F7-Fb1–210, or GE-8019 and CH-F7-Fb1–210, consistently presented moderate to high resistance. Notably, only the local cultivars “Sariq olma” (SS11) and “Besh barmoq” (XK10) harbored all three resistance loci, and phenotypic evaluation likewise revealed these accessions as the most highly resistant to fire blight. This finding clearly demonstrates the cumulative effect of multiple resistance loci, supporting the notion that pyramiding favorable alleles represents a promising strategy to achieve durable and broad-spectrum resistance against fire blight. Similar trends have been reported in previous studies, where the combination of multiple resistance loci conferred stronger and more stable resistance than single-locus effects [14,33].

Comparing results with other germplasm collections, the high PM resistance frequency in Uzbek old varieties (68–88% across regions) is consistent with findings in old German varieties [13]. The co-localization of Pl-1 and Pl-2 on chromosome 12 [28] likely explains the high co-frequency of both alleles in Karakalpakstan accessions, as linked inheritance maintains their coupling phase across generations of open pollination.

From a practical breeding perspective, the six accessions (Atlas olma (SS03), Turkish (SS04), Xuboni (SS10), Krasniy jeleznyak (KB01), Shoyi olma (KH16), Besh barmoq (XK10)) identified as carrying markers for all three diseases represent high-priority candidates for use as donor parents in resistance breeding crosses. A marker-assisted backcross (MAB) strategy could be employed to introgress multiple resistance loci into elite cultivar backgrounds while minimising linkage drag using background selection with neutral markers.

Several limitations of this study should be acknowledged: (i) phenotypic evaluations were conducted under natural field conditions with variable disease pressure across farms and seasons; (ii) standard resistant and susceptible control genotypes were not included for marker validation; (iii) the marker panel covers a subset of known resistance loci (additional loci such as *Rvi4, Rvi15*, and PM QTLs on LG2 and LG3 may have gone undetected); (iv) The absence of the AL07 resistance allele in all accessions may reflect primer-template mismatch arising from sequence divergence between the primer design template (*M. floribunda* 821) and Uzbek apple varieties. AL07 primer specificity in Uzbek germplasm requires sequencing confirmation; (v) Standard reference genotypes (e.g., *M. floribunda* 821 as positive *Rvi6* control, known susceptible cultivars as negative controls) were not included in the present study, since there are not available in local collections. This represents a methodological limitation; validated reference controls will be included in future studies.

## Conclusions

This study presents the first comprehensive molecular evaluation of resistance-associated alleles for fire blight, powdery mildew, and apple scab across 109 indigenous Uzbek apple varieties. Key findings are: (i) substantial genetic variation in resistance-associated loci exists across Uzbek apple germplasm, with regional differentiation reflecting geographic origin and historical selection; (ii) six multi-disease-resistant accessions (Atlas olma, Turkish, Xuboni, Krasniy jeleznyak, Shoyi olma, Besh barmoq) were identified as priority breeding donors; (iii) the AL07/AM19 discrepancy warrants follow-up sequencing; (iv) fire blight resistance remains limited in frequency, emphasizing the need for allele pyramiding strategies.

The resistance-associated markers used in this study indicate the presence of resistance alleles and should be regarded as tools for prioritizing accessions for more detailed phenotypic evaluation, not as standalone criteria for resistance designation. Durable resistance requires validated functional confirmation through controlled inoculation experiments.

Future research directions include: (i) whole-genome sequencing or dense SNP genotyping for comprehensive resistance locus characterization; (ii) controlled inoculation experiments to validate resistance of identified accessions; (iii) inclusion of genome-wide SSR markers for pedigree and population structure analysis; (iv) marker pyramiding in breeding crosses using identified multi-resistant donors.

## Supporting information

S1 Table List of apple genotypes (*Malus domestica*, *M. sieversii*, and *M. niedzwetzkyana*) sampled in Uzbekistan, including variety name, geographic origin, farm/owner. (DOCX)

S1 Fig PCR amplification profile of the AE10–375 marker associated with fire blight resistance in local apple varieties. M100: 100 bp DNA ladder. The expected amplicon size is 375 bp.
(TIF)

S2 Fig PCR amplification profile of the AT20–450 marker associated with field resistance to powdery mildew in local apple varieties. KH01–19 represent plant DNA samples. M100: 100 bp DNA ladder. The expected amplicon size of the AT20–450 marker is 450–500 bp.
(TIF)

## Abbreviations

Tris-HCl: Tris(hydroxymethyl)aminomethane hydrochloride buffer
KCl: Potassium chloride
MgCl_2_: Magnesium chloride
dNTPs: Deoxynucleotide triphosphates
PCR: Polymerase chain reaction
TBE: Tris–borate–EDTA buffer used for agarose gel electrophoresis
NEB: New England Biolabs
SCAR: Sequence-characterized amplified region marker
STS: Sequence-tagged site marker
PCA: Principal component analysis
PM: powdery mildew
AS: apple scab
FB: fire blight (FB)
