# Supplementary material for: Marker-assisted screening of resistance to fire blight, powdery mildew, and apple scab in local apple varieties from Uzbekistan
Source: PLoS One. 2026 Sep 15;21(9):e0357988. doi: 10.1371/journal.pone.0357988 (PMC13577343; doi:10.1371/journal.pone.0357988)
Supplement: Supplementary file 1 [file pone.0357988.s001.docx]

**Supplementary Table 1.** List of apple genotypes (*Malus domestica*, *M. sieversii*, and *M. niedzwetzkyana*) sampled in Uzbekistan, including variety name, geographic origin, farm/owner.

| **№** | **Sample number (code)** | **Taxon** | **Variety** | **Region** | **District** | **Farm/owner names** |
| --- | --- | --- | --- | --- | --- | --- |
| 1 | Skb 01 | *Malus domestica* | Besh yulduz | Surkhandarya | Sherabad | Khujanazarov S. |
| 2 | Skb 02 | *Malus domestica* | Atlas | Surkhandarya | Sherabad | Yeranov U. |
| 3 | Skb 03 | *Malus sieversii* | Qizil olma | Surkhandarya | Sherabad | Choriyev A. |
| 4 | Skb 04 | *Malus sieversii* | Oq olma | Surkhandarya | Sherabad | Uroqova M. |
| 5 | Skb 05 | *Malus sieversii* | Qizil olma | Surkhandarya | Sherabad | Davlatov N. |
| 6 | Skb 06 | *Malus sieversii* | Qizil olma | Surkhandarya | Sherabad | Tajiyev R. |
| 7 | Skb 07 | *Malus sieversii* | Oq olma | Surkhandarya | Sherabad | Abdilov A. |
| 8 | Skb 08 | *Malus sieversii* | Renet Semerenko | Surkhandarya | Sherabad | Abdulov N. |
| 9 | Skb 09 | *Malus sieversii* | Qizil olma | Surkhandarya | Sherabad | Juraeva M. |
| 10 | Skb 10 | *Malus sieversii* | Qizil olma | Surkhandarya | Sherabad | Rahimov Yu. |
| 11 | Sko 01 | *Malus sieversii* | Qizil olma (Red apple) | Surkhandarya | Sherabad | Turoqov P. |
| 12 | Sko 02 | *Malus sieversii* | Qizil olma (Red apple) | Surkhandarya | Sherabad | Almuradov S. |
| 13 | Sko 03 | *Malus sieversii* | Qizil olma (Red apple) | Surkhandarya | Sherabad | Norkulov B. |
| 14 | Sko 04 | *Malus sieversii* | Qizil olma (Red apple) | Surkhandarya | Sherabad | Mamasayidov Sh. |
| 15 | Sko 05 | *Malus domestica* | Renet Semerenko | Surkhandarya | Sherabad | Abdusamatov A. |
| 16 | Sko 06 | *Malus sieversii* | Oq olma | Surkhandarya | Sherabad | Hayitov O. |
| 17 | Sko 07 | *Malus domestica* | Besh yulduz | Surkhandarya | Sherabad | Hayitov Z. |
| 18 | Sko 08 | *Malus sieversii* | Oq olma | Surkhandarya | Sherabad | Ashurov J. |
| 19 | Sko 09 | *Malus sieversii* | Oq olma | Surkhandarya | Sherabad | Shamurodov Sh. |
| 20 | Sko 10 | *Malus sieversii* | Oq olma | Surkhandarya | Sherabad | Hayitov E. |
| 21 | Sko 11 | *Malus domestica* | Besh yulduz | Surkhandarya | Sherabad | Gulsara |
| 22 | Sko 12 | *Malus sieversii* | Oq olma | Surkhandarya | Sherabad | Ashurov M. |
| 23 | Ssh 01 | *Malus domestica* | Renet Semerenko | Surkhandarya | Sherabad | Shofiyev I. |
| 24 | Ssh 02 | *Malus sieversii* | Oq olma | Surkhandarya | Sherabad | Abraev J. |
| 25 | Ssh 03 | *Malus domestica* | Besh yulduz | Surkhandarya | Sherabad | Abraev Sh. |
| 26 | Ssh 04 | *Malus sieversii* | Qizil olma | Surkhandarya | Sherabad | Abraev G. |
| 27 | Ssh 05 | *Malus domestica* | Besh yulduz | Surkhandarya | Sherabad | Abraev O. |
| 28 | Ssh 06 | *Malus domestica* | Renet Semerenko | Surkhandarya | Sherabad | Tursunova M. |
| 29 | Ssh 07 | *Malus sieversii* | Oq olma | Surkhandarya | Sherabad | Muslimkulov B. |
| 30 | Ssh 08 | *Malus domestica* | Besh yulduz | Surkhandarya | Sherabad | Abraev M. |
| 31 | SD01 | *Malus domestica* | Mayskiy | Surkhandarya | Sariasiya | Mukhabatova Nargiza |
| 32 | SD02 | *Malus domestica* | Xuboni | Surkhandarya | Sariasiya | Nazarov Nabi |
| 33 | SD03 | *Malus domestica* | Mayskiy | Surkhandarya | Sariasiya | Nazarov Nabi |
| 34 | SD04 | *Malus domestica* | Xuboni | Surkhandarya | Sariasiya | Jabbarov Nurali |
| 35 | SD05 | *Malus domestica* | Atlas olma | Surkhandarya | Sariasiya | Jabbarov Nurali |
| 36 | SD06 | *Malus domestica* | Mayskiy Qizil | Surkhandarya | Sariasiya | Shapiyev Sattor |
| 37 | SD07 | *Malus domestica* | Eshak olma | Surkhandarya | Sariasiya | Imomov Ulugbek |
| 38 | SD08 | *Malus domestica* | Qizil kechki olma | Surkhandarya | Sariasiya | Imomov Ulugbek |
| 39 | SD09 | *Malus domestica* | Oq olma | Surkhandarya | Sariasiya | Meliboyev Makhkamboy |
| 40 | SS01 | *Malus domestica* | Qirmizak | Surkhandarya | Termez | Yuldashov Isomidin |
| 41 | SS02 | *Malus domestica* | Renet Semerenko | Surkhandarya | Termez | Yuldashov Odil |
| 42 | SS03 | *Malus domestica* | Atlas olma | Surkhandarya | Termez | Yuldashov Odil |
| 43 | SS04 | *Malus domestica* | Turkish | Surkhandarya | Termez | Yuldashov Odil |
| 44 | SS05 | *Malus domestica* | Atlas kechki | Surkhandarya | Termez | Vallamov Rashid |
| 45 | SS06 | *Malus domestica* | Renet Simarenko | Surkhandarya | Termez | Vallamov Rashid |
| 46 | SS07 | *Malus domestica* | Qirmizak | Surkhandarya | Termez | Yuldashov Odil |
| 47 | SS08 | *Malus domestica* | Starkrimson | Surkhandarya | Termez | Yuldashov Odil |
| 48 | SS09 | *Malus niedzwetzkyana* | *Malus niedzwetzkyana* | Surkhandarya | Termez | Sulmonova Nobat |
| 49 | SS10 | *Malus domestica* | Xuboni | Surkhandarya | Termez | Sulmonova Nobat |
| 50 | SS11 | *Malus domestica* | Sariq olma | Surkhandarya | Termez | Sulmonova Nobat |
| 51 | КH 01 | *Malus domestica* | Besh barmoq | Karakalpakstan | Turtkul | Turtkulli Arabovlar |
| 52 | КH 02 | *Malus domestica* | Renet Semerenko | Karakalpakstan | Turtkul | Turtkulli Arabovlar |
| 53 | КH 03 | *Malus domestica* | Samarqandskiy ranniy | Karakalpakstan | Turtkul | Turtkulli Arabovlar |
| 54 | КH 04 | *Malus domestica* | Starkrimson | Karakalpakstan | Turtkul | Turtkulli Arabovlar |
| 55 | КH 05 | *Malus domestica* | Golden delicious | Karakalpakstan | Turtkul | Turtkulli Arabovlar |
| 56 | КH 06 | *Malus domestica* | Jonathan | Karakalpakstan | Turtkul | Turtkulli Arabovlar |
| 57 | КH 07 | *Malus domestica* | Pervenets Samarqanda | Karakalpakstan | Turtkul | Turtkulli Arabovlar |
| 58 | КH 08 | *Malus domestica* | Besh yulduz | Karakalpakstan | Turtkul | Azizxon Shoyimardon |
| 59 | КH 09 | *Malus domestica* | Besh barmoq | Karakalpakstan | Turtkul | Azizxon Shoyimardon |
| 60 | КH 10 | *Malus domestica* | Grimes Golden | Karakalpakstan | Turtkul | Azizxon Shoyimardon |
| 61 | КH 11 | *Malus domestica* | Qand olma kishgi | Karakalpakstan | Turtkul | Azizxon Shoyimardon |
| 62 | КH 12 | *Malus domestica* | Mayskiy | Karakalpakstan | Turtkul | Azizxon Shoyimardon |
| 63 | КH 13 | *Malus domestica* | Red delicious | Karakalpakstan | Turtkul | Azizxon Shoyimardon |
| 64 | КH 14 | *Malus domestica* | Qizil olma urtagi | Karakalpakstan | Turtkul | Azizxon Shoyimardon |
| 65 | КH 15 | *Malus domestica* | Qizil olma | Karakalpakstan | Turtkul | Azizxon Shoyimardon |
| 66 | КH 16 | *Malus domestica* | Shoyi olma | Karakalpakstan | Turtkul | Azizxon Shoyimardon |
| 67 | КH 17 | *Malus domestica* | Tosh olma | Karakalpakstan | Turtkul | Azizxon Shoyimardon |
| 68 | КH 18 | *Malus domestica* | Qand olma | Karakalpakstan | Turtkul | Azizxon Shoyimardon |
| 69 | КH 19 | *Malus domestica* | Krasniy jeleznyak | Karakalpakstan | Turtkul | Azizxon Shoyimardon |
| 70 | ХК 01 | *Malus domestica* | Karvak qutir olma | Khorezm | Khazarasp | Madina Ulmas Shirin |
| 71 | ХК 02 | *Malus domestica* | Avgustovskiy | Khorezm | Khazarasp | Shirin Ulmas Madina |
| 72 | ХК 03 | *Malus domestica* | Ruxi janon | Khorezm | Khazarasp | Shirin Ulmas Madina |
| 73 | ХК 04 | *Malus domestica* | Qand olma | Khorezm | Khazarasp | Shirin Ulmas Madina |
| 74 | ХК 05 | *Malus domestica* | Karvak olma | Khorezm | Khazarasp | Shirin Ulmas Madina |
| 75 | ХК 06 | *Malus domestica* | Qutir qand olma | Khorezm | Khazarasp | Shirin Ulmas Madina |
| 76 | ХК 07 | *Malus domestica* | Jeleznyak | Khorezm | Khazarasp | Shirin Ulmas Madina |
| 77 | ХК 08 | *Malus domestica* | Muz olma | Khorezm | Khazarasp | Shirin Ulmas Madina |
| 78 | ХК 09 | *Malus domestica* | Renet Semerenko | Khorezm | Khazarasp | Shirin Ulmas Madina |
| 79 | ХК 10 | *Malus domestica* | Besh barmoq | Khorezm | Khazarasp | Shirin Ulmas Madina |
| 80 | ХК 11 | *Malus domestica* | Red delicious | Khorezm | Khazarasp | Shirin Ulmas Madina |
| 81 | XS 01 | *Malus domestica* | Besh barmoq | Khorezm | Khanka | Shirin Ulmas Madina |
| 82 | XS 02 | *Malus domestica* | Grimes Golden | Khorezm | Khanka | Sobirjon Yusup |
| 83 | XS 03 | *Malus domestica* | So’x go’zali | Khorezm | Khanka | Sobirjon Yusup |
| 84 | XS 04 | *Malus domestica* | R.Semerenko | Khorezm | Khanka | Sobirjon Yusup |
| 85 | XS 05 | *Malus domestica* | Qizil sho’r olma | Khorezm | Khanka | Sobirjon Yusup |
| 86 | XS 06 | *Malus domestica* | Golden delicious | Khorezm | Khanka | Sobirjon Yusup |
| 87 | XS 07 | *Malus domestica* | Yozgi olma | Khorezm | Khanka | Sobirjon Yusup |
| 88 | XS 08 | *Malus domestica* | Krasniy jeleznyak | Khorezm | Khanka | Sobirjon Yusup |
| 89 | XS 09 | *Malus domestica* | Mayskiy | Khorezm | Khanka | Sobirjon Yusup |
| 90 | XS 10 | *Malus domestica* | Qizil olma | Khorezm | Khanka | Sobirjon Yusup |
| 91 | XS 11 | *Malus domestica* | Xazaraspskiy letniy | Khorezm | Khanka | Sobirjon Yusup |
| 92 | XS 12 | *Malus domestica* | Qand olma kishgi | Khorezm | Khanka | Sobirjon Yusup |
| 93 | XS 13 | *Malus domestica* | Qand olma | Khorezm | Khanka | Durumov Pahlavon |
| 94 | XS 14 | *Malus domestica* | Muz olma | Khorezm | Khanka | Durumov Pahlavon |
| 95 | XS 15 | *Malus domestica* | Xivinskiy beshbaroq | Khorezm | Khanka | Sobirjon Yusup |
| 96 | KБ 01 | *Malus domestica* | Krasniy jeleznyak | Karakalpakstan | Ellikkala | Mansurbek Makhkam |
| 97 | KБ 02 | *Malus domestica* | Jonathan | Karakalpakstan | Ellikkala | Mansurbek Makhkam |
| 98 | KБ 03 | *Malus domestica* | Yozgi olma | Karakalpakstan | Ellikkala | Mansurbek Makhkam |
| 99 | KБ 04 | *Malus domestica* | Starkrimson | Karakalpakstan | Ellikkala | Mansurbek Makhkam |
| 100 | KБ 05 | *Malus domestica* | Xazaraspskiy letniy | Karakalpakstan | Ellikkala | Mansurbek Makhkam |
| 101 | KБ 06 | *Malus domestica* | Xazaraspskiy zimniy | Karakalpakstan | Ellikkala | Mansurbek Makhkam |
| 102 | KБ 07 | *Malus domestica* | Shoyi olma | Karakalpakstan | Ellikkala | Mansurbek Makhkam |
| 103 | KБ 08 | *Malus domestica* | Besh yulduz | Karakalpakstan | Ellikkala | Mansurbek Makhkam |
| 104 | KБ 09 | *Malus domestica* | Golden delicious | Karakalpakstan | Ellikkala | Mansurbek Makhkam |
| 105 | KБ 10 | *Malus niedzwetzkyana* | *Malus niedzwetzkyana* | Karakalpakstan | Ellikkala | Mansurbek Makhkam |
| 106 | KБ 11 | *Malus domestica* | Rozemary white | Karakalpakstan | Ellikkala | Xushnudbek mevasi |
| 107 | KБ 12 | *Malus domestica* | Besh barmoq | Karakalpakstan | Ellikkala | Xushnudbek mevasi |
| 108 | KБ 13 | *Malus domestica* | Yanar | Karakalpakstan | Ellikkala | Xushnudbek mevasi |
| 109 | KБ 14 | *Malus domestica* | Qizil olma (Red apple) | Karakalpakstan | Ellikkala | Bogbon kuvonchi |
